# Transcriptomic Response of White Lupin Roots to Short-Term Sucrose Treatment

**DOI:** 10.3390/plants14030381

**Published:** 2025-01-26

**Authors:** Proyasha Roy, Shrey Sethi, James New, Kristina Mae Lorilla, Karen Maleski, Allan Ancheta, Claudia Uhde-Stone

**Affiliations:** 1 Department of Biological Sciences, California State University, East Bay, Hayward, CA 94542, USA; proy5@horizon.csueastbay.edu (P.R.); jnew@horizon.csueastbay.edu (J.N.);; 2 College of Health Sciences, California Northstate University, Rancho Cordova, CA 95670, USA; allan.ancheta@cnsu.edu

**Keywords:** crosstalk, nutrient deficiency, sucrose, transcriptomics

## Abstract

White lupin (*Lupinus albus*) has become a model plant for understanding plant adaptations to phosphorus (P) and iron (Fe) deficiency, two major limiting factors for plant productivity. In response to both nutrient deficiencies, white lupin forms cluster roots, bottle-brush-like root structures that aid in P and Fe acquisition from soil. While the cluster root function is well-studied, not much is known about the signaling pathways involved in sensing and responding to a P and Fe deficiency. Sucrose has been identified as a long-distance signal sent in increased concentrations from shoot to root in response to both a P and Fe deficiency. Thus, sucrose plays a dual role both as a signal and as a major source of energy for the root. To unravel the responses to sucrose as a signal, we performed an Illumina paired-end cDNA sequencing of white lupin roots treated with sucrose for 20, 40 or 80 min, compared to untreated controls (0 min). We identified 634 up-regulated and 956 down-regulated genes in response to sucrose. Twenty minutes of sucrose treatment showed the most responses, with the ethylene-activated signaling pathway as the most enriched Gene Ontology (GO) category. The number of up-regulated genes decreased at 40 min and 80 min, and protein dephosphorylation became the most enriched category. Taken together, our findings indicate active responses to sucrose as a signal at 20 min after a sucrose addition, but fewer responses and a potential resetting of signal transduction pathways by the dephosphorylation of proteins at 40 and 80 min.

## 1. Introduction

Phosphorus (P) and iron (Fe) are essential plant nutrients, usually quite abundant in soil, but often in forms that are unavailable for uptake by plants [1,2]. As a result, phosphate (P_i_) and Fe deficiency are causing global crop yield losses. Applying more P_i_ fertilizer is not a sustainable solution, because rock phosphate fertilizer is a finite resource that we are currently depleting at an alarming rate [3,4,5].

The crop plant white lupin (*Lupinus albus*) is exceptionally well-adapted to nutrient deficiencies, and has become an illuminating model for the study of plant adaptations to P_i_ and Fe deficiency [6,7]. Under these deficiencies, white lupin forms cluster roots, specialized roots that resemble bottle brushes, which allow white lupin to acquire P_i_ and Fe unavailable to most other plants [6,8]. The function of cluster roots as adaptations to a P_i_ and Fe deficiency has been thoroughly studied using physiological approaches [9,10,11,12,13] and transcriptomics [7,14,15,16,17].

The signaling pathways involved in sensing a P_i_ and Fe deficiency and eliciting responses are, however, not yet well-understood. Split-root experiments in white lupin revealed that a P_i_ deficiency is primarily sensed in the shoot and communicated to the root [18]. Sucrose and specific miRNAs, transported from shoot to root in response to a P_i_ and Fe deficiency, appear to serve as major long-distance signals [19,20,21]. Among the best-studied nutrient-stress-responsive miRNA is miR399. Under a P_i_ deficiency, miR399 cleaves the mRNA of *PHO_2_*, which codes for a ubiquitin E2-conjugating enzyme acting as an inhibitor of the phosphate starvation response [22,23,24,25,26]. In white lupin, miR399 is induced by a P_i_ deficiency, but darkness or stem girdling prevents miR399 induction, suggesting that sucrose is required for miR399 long-distance signaling [27].

Interestingly, sucrose added to the growth medium can induce cluster root formation in white lupin even under sufficient P_i_ and Fe availability [28,29]. However, it is unclear if these sucrose-induced cluster roots are functional. Zhou et al. (2008) [29] reported that the expression of certain P_i_-responsive genes, including *L. albus* phosphate transporter 1 (*LaPT1*), was increased by a combination of a P_i_ limitation and sucrose addition, while the expression of *L. albus* Secreted Acid Phosphatase (*LaSAP*) was induced by sucrose independently of the P_i_ supply [29]. A later study by Wang et al. (2015) confirmed that externally added sucrose triggers cluster root formation in white lupin, but reported that the expressions of P_i_-responsive genes, such as *LaSAP*, were not up-regulated in sucrose-induced cluster roots [28]. These findings suggest the involvement of signals other than sucrose in the regulation of cluster root function. A possible candidate for regulating cluster root function is the disaccharide trehalose, which has recently been shown to be involved in both the formation and function of cluster roots [30].

Interestingly, sucrose appears to act as a long-distance signal not only for a P_i_ deficiency [21,31], but also for other nutrient deficiencies. Lin et al. identified sucrose as a signal involved in Fe deficiency [20]. A sucrose-over-accumulating Arabidopsis mutant (hsp1) revealed that sucrose can act as a common regulator in responses to several different nutrient deficiencies, including P_i_, nitrogen (N), and potassium (K) deficiencies [32]. Sucrose as a shared signal to various nutrient deficiencies may, in part, explain why plant responses to nutrient deficiencies tend to overlap [7], a phenomenon often referred to as crosstalk [33].

The question remains how the increased amounts of sucrose that are sent from the shoot to the root trigger nutrient deficiency responses. Studies in Arabidopsis have revealed that sucrose can regulate gene expression directly by the activation of transcription factors, namely, MYB75 and WRKY20 [34,35]. Sucrose non-fermenting-1-related protein kinases (SnRKs) have been suggested to serve both as sugar transporters and sugar sensors [36], but how exactly cells receive the sucrose signal requires further study. Sucrose signaling appears to integrate signals from multiple pathways to regulate plant stress responses. Soybean, in response to short-term external sucrose, activated hormonal responses, including auxin, jasmonic acid, and salicylic acid, as well as reactive oxygen species (ROS), and calcium signaling [37]. Our recent Nanopore sequencing study in white lupin responses at very early timepoints (10, 15, and 20 min) revealed auxin and gibberellin responses as early as 10 min, and ethylene responses at 20 min of sucrose treatment.

Despite the progress in understanding plant responses to nutrient stress, the underlying mechanism and timeline of sucrose signaling are not yet well-understood [35,38]. In the current study, an RNA-seq analysis has been applied to identify the differential gene expression in white lupin in response to sucrose added directly to the root for 20, 40, and 80 min, compared to the untreated control (0 min). The objective of this research was to reveal early responses to sucrose in roots to identify the main players of the sucrose signaling network, and to compare findings with even earlier timepoints (10, 15, and 20 min) in white lupin [39] and similar research (20 and 40 min) in soybean [37]. The information obtained by unraveling sucrose signaling in plants may prove useful for developing crop plants that are better adapted to a low nutrient availability.

## 2. Results

### 2.1. RNA-Seq Resulted in 382 Million Paired-End Reads

To mimic sucrose as a signal of a P_i_ and Fe deficiency to the root, we added sucrose directly to white lupin roots in a hydroponic system. We used a concentration of 10 mM sucrose based on previous studies in white lupin, which showed that the formation of typical cluster roots (a response to a P_i_ and Fe deficiency) can be mimicked by an external sucrose application in a range of 2.5 to 12.5 mM [28]. Based on this research, we selected 10 mM sucrose as a concentration high enough to elicit responses, but not too high to elicit non-physiological responses.

After hydroponic growth for four weeks in nutrient-sufficient conditions, we added sucrose for 0 (control), 20, 40, and 80 min in three biological replications each, for a total of twelve samples. We generated twelve bar-coded cDNA libraries from these samples, then pooled and sequenced these using the Illumina NovaSeq6000 150PE Flow Cell SP platform, which resulted in an initial 527 million total reads. A FastQC quality check revealed an overall very good sequence quality, with average Phred quality scores above 30 even before trimming, except for the first two and last three nucleotides. A quality check after trimming showed Phred quality scores averaging 35 for all positions, and a complete adaptor removal, and revealed a total number of paired sequences (150 bp each) of 382 million.

Because there are currently two reference genomes available for white lupin [40,41], we mapped our RNA-seq data to both reference genomes, using HiSat2 [42], then assembled transcripts with StringTie [43]. The mapping rates to both reference genomes are almost identical for both reference genomes (Table 1). We next used GFFCompare [44] to assess the mapping quality (Table 2). For reference genome 1 [41], our approach resulted in a total of 33,390 mapped genes out of 38,255 protein-coding genes (87.3%) and out of 41,385 (80.7%) total genes, plus 1260 potentially novel loci. For reference genome 2 [40], we mapped 31,842 out of 47,603 (66.9%) protein-coding and out of 48,718 (65.4%) total genes, and found 1979 potentially new loci. Because the total number of mapped genes was higher for reference genome 1 (Table 2), we continued our analysis with this reference genome sequenced by Hufnagel et al. (2020) [41].

### 2.2. Short-Term (20 min) Sucrose Exposure Changed Expression of More Genes than Longer Exposure (40 and 80 min)

**Figure 1:**
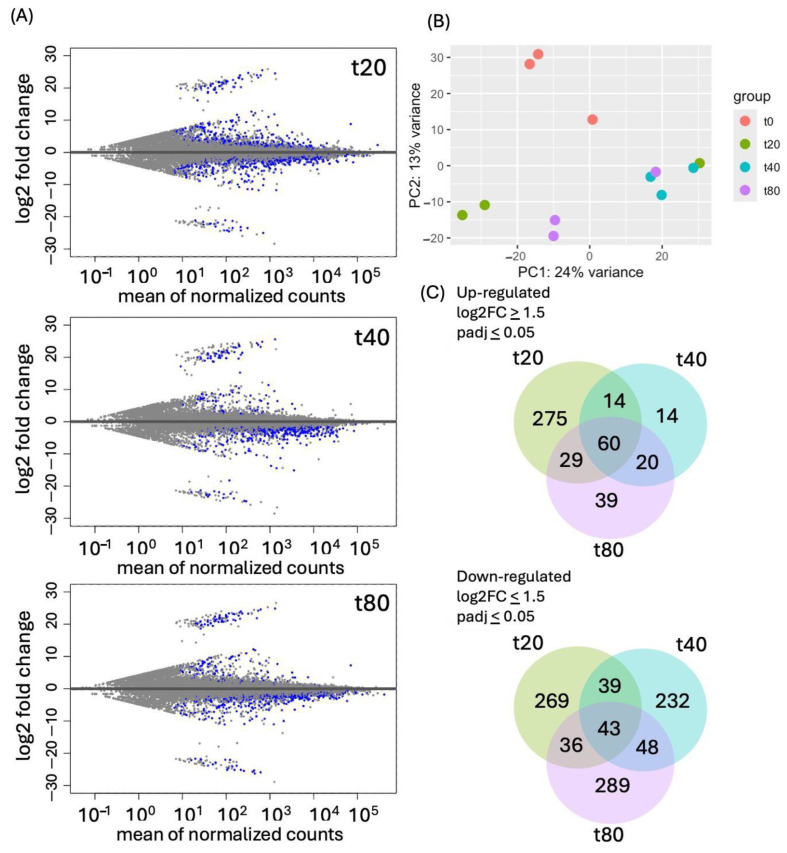
(**A**). MA (mean average) plot of log2 FC against normalized sequence counts at 20, 40, and 80 min (t20, t40, and t80) of sucrose treatment, each compared to t0 (no-sucrose control). Blue dots indicate values of padj (adjusted *p*-value) ≤ 0.01 in the DESeq2 gene expression analysis, while grey dots indicate padj values > 0.01. (**B**) PCA plot of the 100 most up-regulated genes in three biological replications shows differences between sucrose-treated samples versus control, but also reveals some overlap between 20 and 40 min, and 40 and 80 min of sucrose treatment. (**C**) Venn diagram of genes that were significantly up-regulated (**upper panel**) or down-regulated (**lower panel**) after 20, 40, or 80 min of sucrose exposure.

We identified differentially expressed genes with DESeq2 [45] using the time-course option, and also extracted normalized expression data (FPKM, fragment per kilobase, and million) from our HiSat2 data using Ballgown [43,46]. MA (mean average) plots were used to visualize the log2 fold change (FC) against normalized sequence counts at 20, 40, and 80 min of sucrose exposure, each compared to 0 min (control), revealing significantly up- and down-regulated genes at all three timepoints (Figure 1A).

A principal component analysis (PCA) plot (Figure 1B) displays the differences in gene expression between the control and the sucrose-treated samples, but also shows some overlap of t40 with both t20 and t80, revealing some variability between the timeline of responses in biological replications. The overlap between sucrose responses at 20, 40 and 80 min is also noticeable in the Venn diagrams (Figure 1C), which represent the total number of up- and down-regulated genes at the three timepoints. We identified a total of 634 genes that were up-regulated and 956 that were down-regulated. For up-regulated genes, t40 and t80 show more shared than unique up-regulated genes, with a total of 60 up-regulated genes shared at all three timepoints of sucrose treatment.

### 2.3. Several Hormone-Responsive Genes and Transcription Factors Are Expressed at 20 min of Sucrose Treatment

A DeSeq2 time-course analysis to identify differential gene expression gains strength from the availability of similar timepoints, but we noticed considerable variation among fragment per kilobase per million (FPKM) values when focusing on a single timepoint. Thus, we performed a *t*-test on the FPKM values of the three biological replications and used the resulting *p*-value as an additional filter when looking at individual timepoints (indicated as the *t*-test *p*-value).

**Figure 2:**
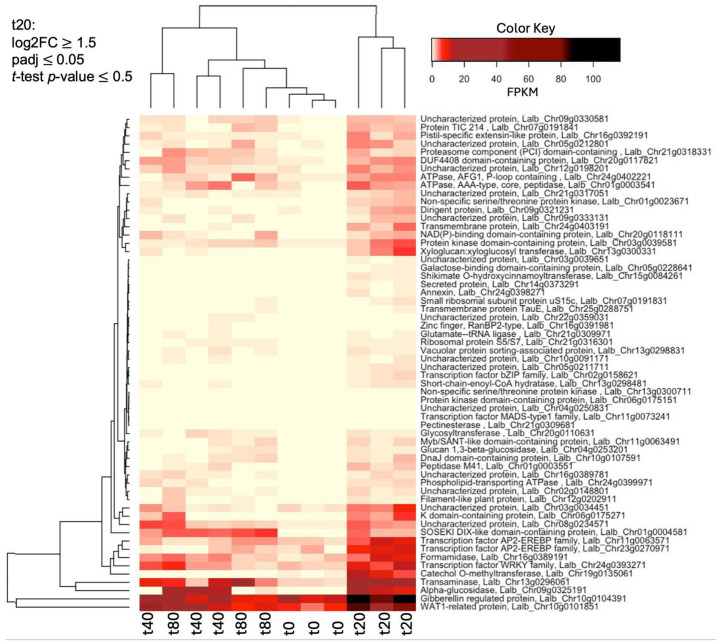
Heatmap of the 60 genes most up-regulated (log2FC ≥ 1.5, padj ≤ 0.05, *t*-test *p*-value ≤ 0.05) at t20 (20 min of sucrose treatment), compared to the untreated control (0 min). Shown are fragments per kilobase per million (FPKM). The raw data for this heatmap can be found in Table S1.

A heatmap of the 60 most up-regulated genes at 20 min of sucrose treatment (Figure 2), compared to the untreated control (0 min), reveals the up-regulation of several transcription factors and hormone-related genes, including WAT-1 (a vacuolar auxin transporter), a gibberellin-regulated gene, and two AP2-EREB2 ethylene response factors, indicating the importance of these plant hormones at the early stage of sucrose responses.

While there are 275 genes that are specifically up-regulated at t20, most of them are still somewhat up-regulated at t40 and t80, but do not reach the log2 threshold of ≥ 1.5. To identify genes that are ONLY up-regulated at t20, but not at t40 and t80, we added a filter of log2FC ≤ 0.5 for t40 and t80. Applying this filter identified 20 genes that were up-regulated ONLY at 20 min (log2FC ≥ 1.5, padj ≤ 0.05, *t*-test *p*-value ≤ 0.05) but not at 40 and 80 min (log2FC ≤ 0.5). Among these 20 genes, we noticed a high presence of transcription factors (7 out of 20; Table 3).

**Figure 3:**
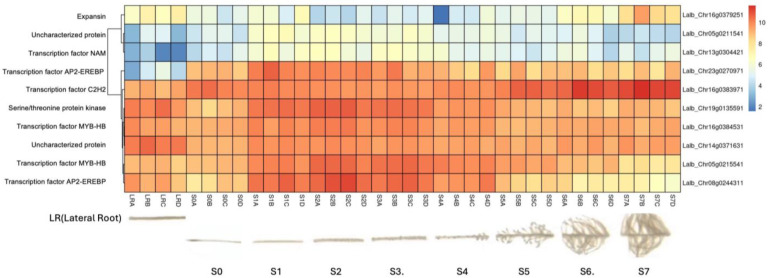
Hierarchical clustering of selected genes that were up-regulated in response to sucrose ONLY at t20, using publicly available gene expression data (https://www.whitelupin.fr/Transcriptomic.html accessed on 24 August 2024). The sections indicate root sections starting from the tip (S0) to visible cluster roots (S4–S7); the letters after the section numbers denote four biological replications. The colors of the heatmap indicate log2-transformed normalized reads. The raw data for this heatmap can be found in Table S2.

Because the master regulators that initiate cluster root development have not been identified and cluster root formation can be induced by an external sucrose application, we were interested in whether any of the genes up-regulated in response to sucrose are also up-regulated during cluster root formation. When we analyzed the 20 genes that were specifically up-regulated at 20 min of sucrose treatment for expression during cluster root development, we found 10 genes—mostly transcription factors—with an induced expression before the emergence of visible cluster roots (Figure 3), making these interesting candidates for the search of key regulators of cluster root development, while two genes (expansin and C2H2 transcription factor) showed up-regulation in developed cluster roots.

### 2.4. Small GTPases and a WRKY Transcription Factor Are Among the Most Up-Regulated Genes in Response to Sucrose at All Three Timepoints

**Figure 4:**
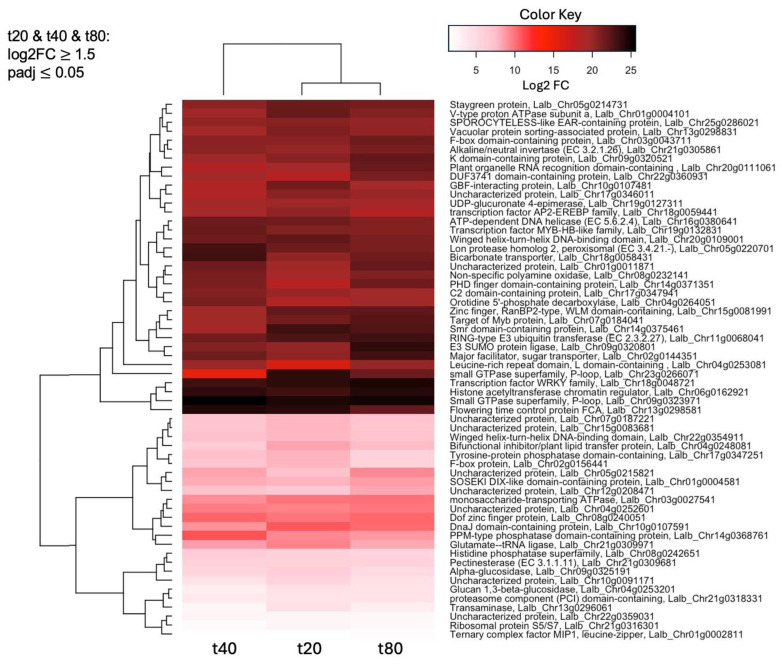
Heatmap of the 60 genes up-regulated (log2FC ≥ 1.5, padj < 0.05) at all three timepoints (20, 40, and 80 min) of sucrose treatment, compared to the untreated control (0 min). Shown are log2FC (fold change). The raw data for this heatmap can be found in Table S3.

The heatmap in Figure 4 gives an overview of the differential expression (shown as log2FC) for the 60 genes that were up-regulated (log2FC ≥ 1.5, padj ≤ 0.05) at all timepoints of sucrose treatment. Standing out as particularly up-regulated at all timepoints are a WRKY transcription factor, a histone acetyltransferase chromatin regulator, a protein belonging to the small GTPase superfamily, and a flowering time control protein. Table 4 gives more detail on the top 30 up-regulated genes at all timepoints, including potential functions, which include protein trafficking and ubiquitination, and the regulation of gene expression, transport, and sugar metabolism.

### 2.5. Gene Ontology (GO) Analysis Reveals Early Enrichment of Ethylene-Activated Signaling

**Figure 5:**
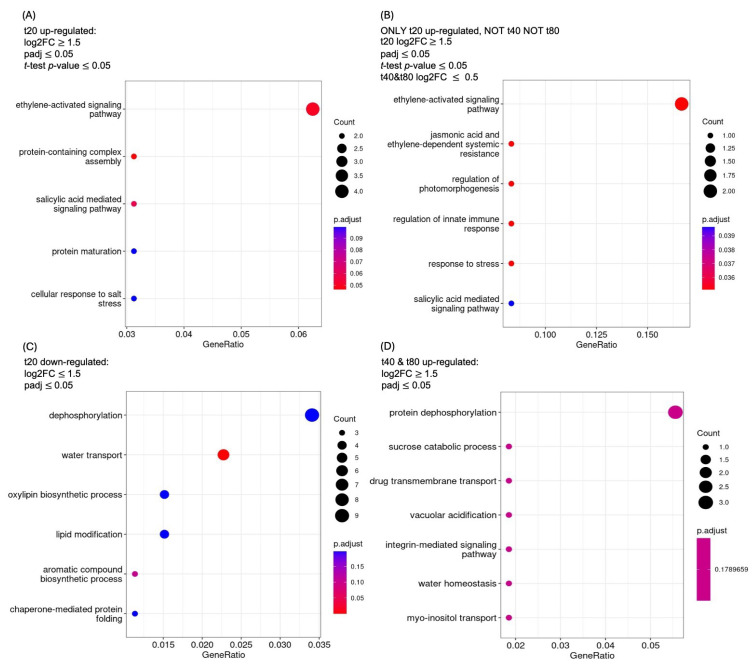
GO enriched categories of biological functions. GO enrichment of (**A**) genes up-regulated at 20 min of sucrose treatment, (**B**) genes ONLY up-regulated at 20 min, (**C**) genes down-regulated at t20, and (**D**) genes up-regulated at 40 AND 80 min of sucrose exposure.

A GO enrichment analysis of genes up-regulated at 20 min of sucrose treatment (Figure 5A) shows the “ethylene-activated signaling pathway” as the most enriched category, followed by the “protein-containing complex assembly” and “salicylic acid mediated signaling pathway”. Because we were also interested in genes that were ONLY up-regulated at 20 min of sucrose treatment (log2FC≤ 0.5 for t40 and t80), we looked for GO enrichment in this group as well (Figure 5B). The GO categories enriched in both sets (t20 and t20 ONLY) are the “ethylene-activated signaling pathway” and the “salicylic acid-mediated pathway”. The biotic-stress response-related categories “jasmonic acid and ethylene-dependent systemic resistance” and “regulation of innate immune response” are enriched among genes that are up-regulated at t20 ONLY.

A GO analysis of genes down-regulated at t20 shows dephosphorylation as the most enriched category (Figure 5C). This trend reverses at 40 and 80 min of sucrose exposure, where protein dephosphorylation becomes the most enriched category among up-regulated genes, followed by the “sucrose catabolic process” (Figure 5D).

### 2.6. Promoter Analysis Reveals Enrichment of Putative Transcription Factor-Binding Motifs

We retrieved promoter sequences from the white lupin genome browser [41] for various sets of sucrose-responsive genes. We then looked for promoter motifs that were enriched in one set (the test set) compared to a control set of the same size. Our comparisons included genes up-regulated versus down-regulated at all timepoints, as well as genes up-regulated ONLY at 20 min compared to genes up-regulated at all timepoints, and vice versa, and genes that showed up-regulation during cluster root development (Table 5).

Using a cut-off *p*-value of <10^−5^, we found several putative promoter motifs that showed enrichment. We then used TomTom [47] to identify potential transcription factors binding to these enriched motifs (Table 5). Of special interest among these is the enrichment of a motif similar to the binding motif of transcription factor HHO6, which, according to Uniprot (HHO6_ARATH), may be involved in phosphate signaling in roots, based on its similarity to HRS1 [48]. Also of interest is the enrichment of an AP2/ERF (ethylene-response factor)-binding motif among the 20 genes up-regulated ONLY at t20, compared to genes up-regulated at all timepoints, considering that the ethylene response is an enriched category specifically at 20 min of sucrose treatment.

## 3. Discussion

### 3.1. Reference Genome 1 Is a Good Option for Mapping White Lupin Transcripts

Plants have evolved a coordinated response to a P and Fe deficiency, which involves the increased allocation of C to the root, mainly in the form of sucrose. Increased sucrose allocation optimizes root growth toward a higher root-to-shoot ratio and more lateral root growth [30,38,39]. But sucrose transported from the shoot to the roots can also act as a long-distance signal for both a P [20] and Fe [40] deficiency. We were interested in unraveling the regulatory network that becomes activated in the root in response to a sucrose addition directly to the root. We used white lupin, because of its ability to form cluster roots in response to a P and Fe deficiency, making it a model plant for studying adaptations to these deficiencies.

Our Illumina paired-end sequencing resulted in 382 million paired-end reads. Because two reference genomes of white lupin are available, we mapped our reads to both, in order to give a recommendation on which to use for mapping. Overall, mapping was similarly effective for both genomes, with almost identical mapping rates. Hufnagel et al. identified 41,385 total transcripts in reference genome 1 [41], which is less than the 47,603 identified by Xu et al. in reference genome 2 [40]. However, our mapping matched more genes (33,390 out of 38,255 protein-coding genes; 87.3%) in reference genome 1 than in reference genome 2 (31,842 out of 47,603 protein-coding genes; 66.9%), indicating that reference genome 1 is a good option for mapping white lupin transcripts. Compared to our previous Nanopore-based transcriptomics [39], which had resulted in 21 million reads, corresponding to 24,655 genes (64% of coding genes) mapped to reference genome 1, our current Illumina-based approach mapped 8735 (35%) more genes.

### 3.2. Hormone Responses Are Enriched at 20 min of Sucrose Treatment

Our previous RNA-seq (Illumina paired-end) research in soybean revealed 358 up-regulated genes at 20 min of sucrose, which is very similar to the number of 378 up-regulated genes that we found here. However, at 40 min, the number in soybean increased drastically to 2416 genes, while, in white lupin, the number of up-regulated genes decreased to 108 genes at 40 min and 148 genes at 80 min, with a high overlap (60 genes) that was up-regulated at all three timepoints of sucrose treatment.

A GO enrichment analysis revealed the “ethylene-activated signaling pathway” as the most enriched among genes up-regulated after 20 min of sucrose exposure. This pathway was also enriched at 20 min of sucrose exposure in a previous study of white lupin, in which we compared 10, 15, and 20 min of sucrose responses using Nanopore sequencing. We also found a slight enrichment in the biotic-stress-response-related categories “jasmonic acid and ethylene-dependent systemic resistance” and “regulation of innate immune response” and “salicylic acid-mediated pathway”. Similar biotic stress responses were also enriched in soybean in response to 20 and 40 min of sucrose, though the enrichment has been much higher in soybean [37]. We did not find any enrichment of reactive oxygen species (ROS)- and Ca^2+^-signaling in white lupin, which were enriched in soybean after 40 min of sucrose.

Other enriched responses in our previous study were responses to the plant hormones auxin, gibberellin and brassinosteroids. While these pathways were not enriched categories in our GO analysis, we did find an up-regulation of phytohormone-responsive genes, including gibberellin-responsive genes, auxin-induced protein, and WAT-1, a vacuolar auxin transporter, at 20 min of sucrose treatment, supporting the role of auxin and gibberellin—in addition to ethylene—in white lupin’s early response to sucrose.

### 3.3. Kinases Were Up-Regulated at 20 min of Sucrose Treatment, While Protein Dephosphorylation Is Enriched at 40 and 80 min

The most enriched category among genes up-regulated at 40 and 80 min is protein dephosphorylation. Protein dephosphorylation can play an important part in signal transduction by removing phosphate groups that were added earlier. Because of the enrichment of dephosphorylation at later timepoints of sucrose exposure, we were interested in the ratio of kinases to phosphatases at the three timepoints of sucrose treatment (Table 6). Indeed, the number of up-regulated kinases to phosphatases is much higher at 20 min (17:4; ratio of 4.5) than at 80 min (3:3; ratio of 1), indicating more active protein phosphorylation at earlier timepoints and more dephosphorylation at later timepoints.

### 3.4. Promoter Analysis Reveals Candidate Binding Sites for Sucrose-Responsive Transcription Factors

Transcriptionally co-regulated genes in response to sucrose may share common transcription factor binding motifs in their promoters. We were interested in sets of co-regulated genes that were regulated differently in response to sucrose, such as those up- versus down-regulated at all three timepoints, or up-regulated at t20 ONLY versus up-regulated at all three timepoints. After identifying the most significantly enriched motifs in the promoter regions of these gene sets, we used TomTom to predict possible transcription factors that bind to these [47]. We found, indeed, several sets of motifs enriched in our comparisons (Table 5), indicating that sucrose responsiveness is regulated by sets of different transcription factors. Motifs found include a motif similar to the transcription factors HHO6 and HRS1, which are predicted to be involved in phosphate signaling in roots [48].

Because the key regulator(s) of cluster root development are still unknown, and because external sucrose can induce the formation of cluster roots [28,29], we looked at our sets of up-regulated genes for interesting expression patterns in cluster root development. While we did not see enriched cluster-root specific patterns in most of our gene sets, when we used the 20 genes ONLY up-regulated at 20 min of sucrose, 10 out of 20 showed up-regulation at specific stages of cluster-root development (Figure 3). Most of these were transcription factors that were up-regulated before the emergence of visible cluster roots, making these interesting candidates for regulators of cluster root formation. We compared promoter regions of these 10 genes with the remaining 10 genes that were up-regulated at t20 ONLY, but that did not show any changes of expression during cluster root development. One enriched motif similar to a MADS transcription factor binding motif was found in all 10 test promoters, but in none of the 10 control promoters. Our identification of enriched motifs among promoter regions of sucrose-induced genes and their putative transacting factors provides potential targets for the further dissection of the mechanisms of sucrose signaling and sucrose-induced cluster root formation in white lupin.

### 3.5. Pathway Analysis Summarizes Responses to Sucrose at 20, 40, and 80 min

**Figure 6:**
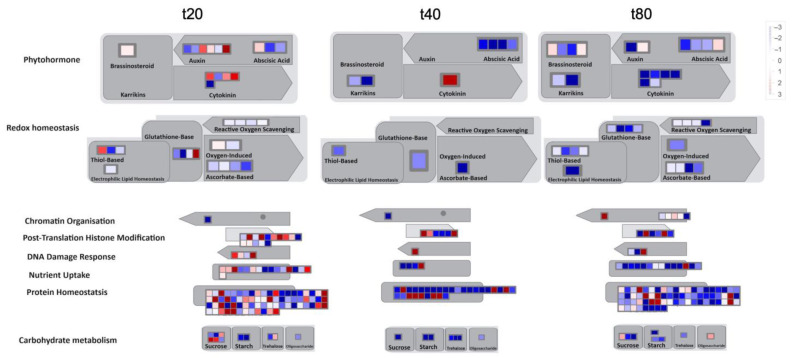
Pathway analysis using MapMan reveals many up-regulated pathways at 20 min of sucrose treatment, but mostly down-regulated pathways at 40 and even more so at 80 min of sucrose exposure. Input are log2FC values with padj ≤ 0.05. Red indicates up-regulated, and blue down-regulated genes.

To obtain an overview of the cellular responses to sucrose, we performed a pathway analysis of differentially expressed genes at 20, 40, and 80 min of sucrose treatment (Figure 6). Several phytohormone pathways are up-regulated at 20 min of sucrose exposure. This finding is consistent with our previous findings in white lupin [39], in which, particularly, genes involved in plant hormone responses to auxin, gibberellin, and ethylene were up-regulated at 10, 15, and/or 20 min of sucrose treatment.

Other pathways that show noticeable changes include histone modifications, nutrient uptake, and protein homeostasis (Figure 6). However, our current study reveals that most inductions of gene expression may be only a short-term response. With more time, a trend of less up-regulation and more down-regulation becomes apparent (40 min), cumulating in more down-regulation of these pathways at 80 min of sucrose treatment.

Recent transcriptomic experiments in the closest algal relatives of land plants, Zygnematophyceae, exposed to light and heat stress, combined with the extensive data mining of stress response experiments in plants, have identified conserved stress hubs that are common to both algae and plants, indicating that these originated before plants moved to land [49].

Indeed, we found some components of these general stress response hubs differentially expressed in our study, including genes related to plant hormones, such as ethylene response factors and abscisic acid (ABA) signaling. Other common stress response genes involving mitogen-activated protein kinases (MAPKs) were represented among differentially expressed genes in our study, though they were not significantly enriched categories.

### 3.6. Sucrose Can Act as Signaling Molecule, but So Can Its Cleavage Products

Sucrose has long been viewed only as a source of energy, but an additional role of sucrose in signaling is emerging [35,38,50]. A main reason for neglecting the possibility of sucrose as a signal was the assumption that sucrose is rapidly metabolized. Two families of sucrose-cleaving enzymes have been identified in plants, invertases and sucrose synthases [51]. Invertases cleave sucrose into glucose and fructose, while sucrose synthases cleave sucrose in the presence of UDP into UDP-glucose (UDP-G) and fructose. Our data showed a gene encoding invertase among the three most up-regulated genes at all three timepoints of sucrose treatment. We were interested in whether the invertase cleaves sucrose outside or inside the cell. A computational analysis of protein localization using Phobius [52] revealed that the invertase is likely anchored in the membrane with a single transmembrane-spanning domain, with the remaining portion of the protein localized in the cytoplasm, indicating the cleaving of sucrose inside the cell, rather than in the apoplast. An ATP-dependent monosaccharide transporter was also among the up-regulated genes at all three timepoints of sucrose treatment, indicating some potential cleaving of the disaccharide sucrose outside of the cell, and actively importing the monosaccharides. Both sucrose cleavage products, glucose and fructose, can also act as signaling molecules, making it possible that some of the observed responses result from glucose and fructose signaling pathways. Glucose is part of the hexokinase signaling pathway [51], while fructose may signal via a pathway involving abscisic acid (ABA) and ethylene [53]. To distinguish these, one could repeat our experiment with glucose or fructose instead of sucrose, and determine which responses are overlapping.

### 3.7. Short-Term Responses to Sucrose and P or Fe Deficiency Display Considerable Overlap

While long-term responses to P and Fe deficiencies have been well-studied in white lupin [7,54], less is known about their short-term effects. A microarray analysis in Arabidopsis compared the gene expression after 1, 2, and 24 h of P deficiency in leaves and roots [55]. Among the earliest responses in roots (1 h) were pectin-related processes, with the up-regulation of pectinesterase and extensins. Similarly, we found pectinesterases, extensin, and other genes involved in cell wall modification among genes up-regulated in response to short-term sucrose, both in this study, and in our previous study at even earlier timepoints (10, 15, and 20 min of sucrose exposure), indicating the importance of cell wall alterations in response to both short-term sucrose exposure and P deficiency.

Other genes up-regulated at 1 h of P deficiency included kinases and transcription factors, such as NAC, WRKY, and ETHYLENE-RESPONSIVE ELEMENT BINDING FACTOR (ERF) family members [55]. The majority of transcription factors up-regulated in response to a short-term P deficiency were involved in ethylene, jasmonic acid, and salicylic acid signaling [55]. These hormone-signaling pathways were also among the enriched pathways in our study of the early sucrose response, showing further overlap between short-term sucrose and P deficiency responses.

An RNA-seq study in soybean in response to 1 h of Fe deficiency identified several kinases and transcription factors, as well as two homologs of the “bifunctional inhibitor/lipid-transfer protein/seed storage 2S albumin superfamily protein” [56]. A protein with this annotation was also up-regulated at all timepoints of sucrose treatment (Figure 4), and up-regulated in our previous study in white lupin at all timepoints of sucrose exposure (10, 15, and 20 min) [39]. Members of this protein family are typically lipid transfer proteins located in the cell wall and involved in key cellular processes, such as cell wall organization, and signal transduction [57]. Interestingly, transcripts up-regulated in response to a short-term Fe deficiency in leaves included SWEET12 [56], a sucrose transporter involved in loading sucrose from leaves into the phloem, supporting the importance of sucrose as a long-distance signal of Fe deficiency that is sent from the shoot to the root. Among the most enriched GO terms in roots under a short-term Fe deficiency (1 h) were “Response to ethylene and other stimuli”, and “Cell wall organization or biogenesis” [56], which overlap with enriched GO terms after short-term sucrose treatment (Figure 4) and P deficiency [55].

In another study of soybean exposed to Fe deficiency for 30, 60, and 120 min [58], many of the most enriched categories were involved in the defense against biotic stress, such “respiratory burst”, “defense response to fungus”, and “systemic acquired resistance, salicylic acid-mediated”. Interestingly, these were also among the most enriched GO terms in response to sucrose (20 and 40 min) in our previous study in soybean [37], but to a lesser extent in white lupin, indicating variations of responses to sucrose among different plant species.

**Figure 7:**
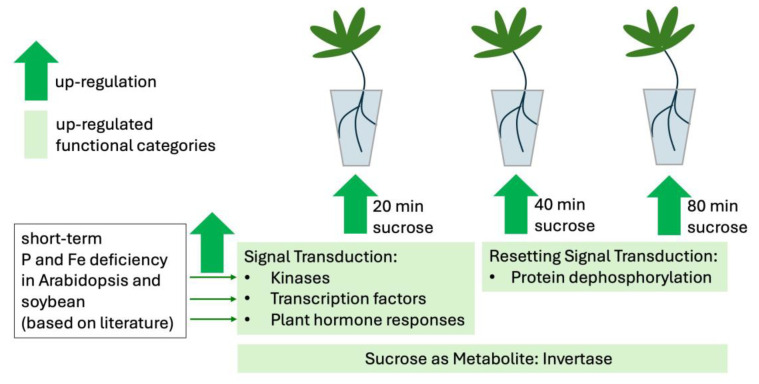
Working model summarizing our findings on short-term responses of roots to sucrose. Kinases, transcription factors, and plant hormone responses are up-regulated after 20 min of sucrose exposure, as well as in response to short-term P and Fe deficiency in Arabidopsis and soybean roots, as revealed in the literature [55,56,58]. However, after 40 and 80 min of sucrose, protein dephosphorylation becomes an enriched category, indicating a possible resetting of signal transduction pathways. A dual role of sucrose also as metabolite is evident by the up-regulation of invertase, an enzyme cleaving sucrose into glucose and fructose, at all three timepoints of sucrose treatment.

Overall, there is considerable overlap between the early responses to sucrose, P deficiency, and Fe deficiency. However, specific P or Fe starvation responses, such as secreted acid phosphatase, or high-affinity P_i_ (inorganic phosphate) or Fe transporters were not up-regulated in our short-term sucrose-response study. This may be due to the short exposure time to sucrose, but it is also possible that sucrose alone does not induce the expression of specific nutrient starvation genes. This possibility is supported by the finding that long-term sucrose treatment triggered cluster root formation in white lupin, but did not induce the up-regulation of known P starvation-induced genes [28]. Figure 7 shows a working model that summarizes our findings of white lupin’s responses to short-term sucrose (as a signal and as a metabolite) and short-term responses to P and Fe deficiency in Arabidopsis and soybean. In future, it will be interesting to compare the gene expression after a longer sucrose exposure (e.g., 12, 24, and 48 h) with the corresponding P and Fe deficiency responses.

## 4. Materials and Methods

### 4.1. Seed Germination, Treatments, and Harvest

*Lupinus albus* cv. Amiga seeds were sterilized by shaking for 3 min in 10% bleach, followed by 6 rinses with autoclaved water. Seeds were half-covered with sterile water and germinated at room temperature for 4 days. Once radicles reached 3 to 5 cm in length, germinated seeds were transferred to containers containing 850 mL Hoagland nutrient solution [59] and were grown for 4 weeks; Hoagland solution was replenished as needed. The growth chamber conditions were maintained at ~21 °C with a cycle of 16 h light and 8 h dark. For sucrose treatment, 8.5 mL of 1 M sucrose (prepared in Hoagland solution) was added directly to the hydroponic solution, for a final concentration of 10 mM sucrose. 

Harvesting occurred at 0 min (control), and after 20, 40, and 80 min of sucrose addition. To enable statistical data analysis, all-timepoints were carried out in 3 biological replications, with each replication consisting of an individual plant. Per plant, about 100 mg of root tip sections (~4–5 cm) were harvested in liquid nitrogen and immediately stored at −80 °C.

### 4.2. RNA Isolation and Quality Check

RNA from frozen root samples was isolated using the RNeasy Plant Mini kit (Qiagen, Valencia, CA, USA). TapeStation (Agilent, Santa Clara, CA, USA) was used to assess both quantity and quality of each sample; only RNA samples with RNA integrity Numbers (RINs) above 8 were used for RNA-seq.

### 4.3. cDNA Library Preparation and RNA-Sequencing

The Stranded mRNA Kit (Illumina, Foster City, CA, USA) was used to convert the extracted RNA to cDNA, following the manufacturer’s instructions. Unique dual barcoding for each cDNA library was carried out using the IDT for Illumina RNA UD Indices Set A (Illumina).

Quantity and quality of cDNA libraries were assessed via TapeStation (Agilent, Santa Clara, CA, USA) and remaining adaptors were cleaned up where necessary using AMPure XP magnetic beads (New England Biolabs, Ipswich, MA, USA) at a ratio of 1 volume DNA to 0.8 volumes of beads. 

Exact quantification, pooling, and sequencing of the 12 barcoded cDNA libraries was performed at QB3 (Institute for Quantitative Biosciences at UC Berkeley). Sequencing of the pooled cDNA libraries was performed on one flowcell lane on the Illumina NovaSeq6000 SP 150PE (paired-end) next-generation sequencing platform.

### 4.4. RNA-Seq Data Analysis

Illumina bcl2fastq2 (v2.20) Conversion Software was used to demultiplex the obtained sequencing data and to convert base call files into FASTQ files. We transferred demultiplexed FastQ files containing 527 million reads from the QB3 server to our storage allocation at the EXPANSE supercomputer housed at San Diego Supercomputer Center (SDSC). We checked sequence quality using FastQC (https://qubeshub.org/resources/fastqc accessed on 15 June 2024). Next, we removed adaptors and any low-quality sequences with TRIMMOMATIC Version 0.32 [60]. Sequence quality was again checked by FastQC to ensure TRIMMOMATIC properly removed all adaptors and low-quality regions. Then, all paired RNA-seq reads were mapped with HiSat2 [42] to the two *Lupinus albus* reference genomes that are currently available [40,41]. Both reference genomes are available at NCBI, as CNRS_Lalb_1.0 (GCA_009771035.1 assembly; submitted 20 December 2019) (https://www.ncbi.nlm.nih.gov/datasets/genome/GCA_009771035.1/ accessed on 20 June 2024) [41] and La_Amiga3.1 (GCA_010261695.1 assembly, submitted 6 February 2020) (https://www.ncbi.nlm.nih.gov/datasets/genome/GCA_010261695.1/ accessed on 20 June 2024) [40]. After initial mapping, we used StringTie [43] for transcript assembly. 

### 4.5. Differential Expression Analysis

We used the prepDE.py3 script (http://ccb.jhu.edu/software/stringtie/dl/prepDE.py3 accessed on accessed on 26 June 2024) to extract two comma-separated value (CSV) files with transcript and gene count information. These files were explored further in RStudio. We used DESeq2 for time course analysis of differential expression [45]. In addition, we extracted the normalized expression data fragment per kilobase and million (FPKM) using Ballgown, [46]. Both packages (DeSeq2 and Ballgown) allow spliced transcriptome assembly for differential expression analysis.

To identify differentially expressed genes (DEGs), we set a threshold of log2FC (fold change) ≥ 1.5 or ≤ 1.5 and an adjusted *p*-value (padj) ≤ 0.05. DESeq2 was also used to generate mean average (MA) and principal component analysis (PCA) plots [45]. Heatmaps of FPKM data were generated by gplots (https://cran.r-project.org/web/packages/gplots/index.html accessed on 12 August 2024). Enrichment analysis of GO terms was performed on DEGs, using the GO term Enrichment tool on the White Lupin Genome browser (https://www.whitelupin.fr/Transcriptomic.html accessed on 24 August 2024) (Hufnagel et al., 2020 [41]).

### 4.6. Promoter Analysis 

In this study, 1 kb representing promoters up to the start codon were extracted in FASTA format from the White Lupin Genome browser for gene sets of interest (https://www.whitelupin.fr/gene_sequence_download.html accessed on 21 November 2024). Promoter analysis was performed using the MEME bioinformatics suite, specifically STREME [61] and TOMTOM [47]. STREME can compare frequency of motifs in one set versus a user-provided control set. In this mode, both sets of promoters must be of the same size. We used default parameters, except the motif size was set to a range between 6 and 15 bp. Significant STREME motifs were then compared with TOMTOM [47] to the Jaspar Core Plants and Arabidopsis collections of known transcription factor binding sites, using a *p*-value cut-off of <5 × 10^−3^.

### 4.7. MapMan Analysis of Metabolic Pathways

MapMan version 3.5.1R2 was downloaded from the MapMan database (https://MapMan.gabipd.org/MapMan accessed on 2 October 2024). The complete set of white lupin protein sequences was obtained in FASTA format from the White Lupin Genome browser (https://www.whitelupin.fr/download.html accessed on 2 October 2024). These sequences served as input for protein function mapping using the Mercator4 v7.0 online tool. The resulting mapping file was subsequently used for pathway visualization in MapMan. Differentially expressed genes (DEGs) were filtered for metabolic pathway analysis using an adjusted *p*-value threshold (padj) ≤ 0.05. The study incorporated gene identification labels and their corresponding log2FC values at three distinct timepoints: 20, 40, and 80 min of sucrose treatment. We selected the “Metabolism_overview” in MapMan to visualize metabolic changes across the experimental timepoints.

## 5. Conclusions

In conclusion, our results indicate that the up-regulation of gene expression in response to sucrose was more pronounced at 20 min than at 40 and 80 min of sucrose treatment. Most notable were the responses to plant hormones, particularly to ethylene. More kinases were up-regulated at 20 min, while protein dephosphorylation became an enriched category at 40 and 80 min of sucrose treatment, indicating a potential signal transduction at 20 min and a resetting of the signal transduction cascade at 40 and 80 min. A promoter analysis revealed several binding sites for potential sucrose-responsive transcription factors. A comparison to short-term responses to a P or Fe deficiency revealed considerable overlap. Together, these results provide potential targets for the further dissection of the mechanisms of sucrose signaling and sucrose-induced cluster root formation in white lupin.

## Figures and Tables

**Table 1:** Number of paired sequences and mapping rates for the twelve cDNA libraries to the two reference genomes currently available for white lupin genome.

cDNA Library, Biological Replicate (rep)	Number of Paired Sequences (in Millions)	Overall Mapping Rate to Reference Genome 1 [41]	Overall Mapping Rate to Reference Genome 2 [40]
Control (t0), rep 1	24.1	86.63%	86.67%
Control (t0), rep 2	27.8	91.64%	91.65%
Control (t0), rep 3	28.3	90.54%	90.58%
20 min sucrose (t20), rep 1	38.1	79.33%	79.42%
20 min sucrose (t20), rep 2	18.8	94.04%	94.26%
20 min sucrose (t20), rep 3	37.4	91.37%	91.38%
40 min sucrose (t40), rep 1	25.8	90.88%	90.91%
40 min sucrose (t40), rep 2	29.4	91.10%	91.14%
40 min sucrose (t40), rep 3	34.4	87.78%	87.81%
80 min sucrose (t40), rep 1	40.7	83.84%	83.87%
80 min sucrose (t40), rep 2	46.2	91.20%	91.22%
80 min sucrose (t40), rep 3	30.5	92.63%	92.67%
Total	381.5	89.25%	89.3%

**Table 2:** Mapping quality as assessed by GFFCompare.

Features	Mapping Statistics for Reference Genome 1 [41]	Mapping Statistics for Reference Genome 2 [40]
Total transcripts in reference genome	41,385 (30,479 multi-exon)	47,603 (38,154 multi-exon)
Mapped genes in our RNA-seq analysis	33,390 (80.7%)	31,842 (66.9%)
Novel exons	6457/25,1260 (2.6%)	12,152/30,6263 (4.0%)
Novel introns	6724/17,9038 (3.8%)	7400/21,8573 (3.4%)
Novel loci	1260/40,617 (3.1%)	1979/48,624 (4.1%)

**Table 3:** 20 genes up-regulated ONLY at 20 min of sucrose exposure, but not at 40 and 80 min, compared to the untreated control (0 min).

Annotation, Gene ID	Putative Function	t20 Log2FC * (padj)	t40 Log2FC ** (padj)	t80 Log2FC ** (padj)
Catechol O-methyltransferase, Lalb_Chr19g0135061	Catalyzing methylation of various phenolic compounds, including lignin monomers	4.4 (3.2 × 10^−08^)	−0.9 (0.9)	−1.7 (0.2)
Shikimate O-hydroxy-cinnamoyl transferase, Lalb_Chr15g0084261	Involved in generating building blocks of lignin	3.3 (4.1 × 10^−02^)	−0.2 (1.0)	0.0 (1.0)
Transcription factor AP2-EREBP family, Lalb_Chr23g0270971	Regulation of gene expression	2.8 (3.0 × 10^−02^)	−1.0 (1.0)	−0.2 (1.0)
Transcription factor MYB-HB-like family, Lalb_Chr05g0215541	Regulation of gene expression	2.1 (2.0 × 10^−06^)	0.0 (1.0)	0.4 (0.7)
Uncharacterized protein, Lalb_Chr05g0211541		2.0 (3.4 × 10^−04^)	0.2 (1.0)	−0.3 (0.8)
Transcription factor AP2-EREBP family, Lalb_Chr08g0244311	Regulation of gene expression	1.9 (2.0 × 10^−02^)	−0.3 (1.0)	0.0 (1.0)
Tetratricopeptide-like helical domain-containing protein, Lalb_Chr13g0300041	Mediates protein interactions	1.9 (4.6 × 10^−02^)	−0.2 (1.0)	0.4 (0.8)
Uncharacterized protein, Lalb_Chr23g0267501		1.8 (4.9 × 10^−02^)	0.3 (1.0)	−0.2 (0.9)
Transcription factor NAM family, Lalb_Chr13g0304421	Regulation of gene expression	1.7 (3.1 × 10^−02^)	−0.6 (1.0)	0.2 (0.9)
RING-type E3 ubiquitin transferase, Lalb_Chr10g0104201	Ubiquitination of proteins	1.7 (3.4 × 10^−02^)	0.3 (1.0)	−1.3 (0.2)
F-box domain-containing protein, Lalb_Chr18g0047021	Ubiquitination of proteins	1.7 (1.3 × 10^−02^)	0.2 (1.0)	0.4 (0.8)
Non-specific serine/threonine protein kinase, Lalb_Chr19g0135591	Signal transduction	1.6 (5.8 × 10^−04^)	0.5 (0.9)	0.4 (0.7)
Transcription factor C2H2 family, Lalb_Chr16g0383971	Regulation of gene expression	1.6 (3.3 × 10^−03^)	0.5 (1.0)	0.1 (1.0)
Uncharacterized protein, Lalb_Chr15g0090181		1.6 (9.7 × 10^−03^)	0.4 (1.0)	0.0 (1.0)
ATPase, AAA-type, Lalb_Chr07g0180441	ATPases associated with diverse cellular activities	1.6 (1.7 × 10^−03^)	0.1 (1.0)	0.1 (0.9)
Uncharacterized protein, Lalb_Chr17g0346531		1.6 (6.4 × 10^−03^)	0.1 (1.0)	0.0 (1.0)
Transcription factor MYB-HB-like family, Lalb_Chr16g0384531	Regulation of gene expression	1.6 (4.6 × 10^−04^)	0.1 (1.0)	0.5 (0.6)
Uncharacterized protein, Lalb_Chr14g0371631		1.6 (2.3 × 10^−03^)	0.1 (1.0)	0.1 (0.9)
Expansin, Lalb_Chr16g0379251	Loosening cell walls for cell expansion	1.5 (3.8 × 10^−02^)	−0.1 (1.0)	−0.1 (0.9)
Transcription factor C3H family, Lalb_Chr22g0350601	Regulation of gene expression	1.5 (2.6 × 10^−02^)	−0.5 (1.0)	−0.4 (0.7)

* log2FC ≥ 1.5, padj ≤ 0.05, *p*-value ≤ 0.05; ** log2FC ≤ 0.5.

**Table 4:** 30 most up-regulated genes (log2FC ≥ 1.5, padj ≤ 0.05) at all three timepoints (20, 40, and 80 min) of sucrose treatment, compared to the untreated control (0 min), sorted for t20.

Gene ID/ Annotation, Gene ID	Putative Function	t20 log2FC (padj)	t40 log2FC (padj)	t80 log2FC (padj)
Small GTPase superfamily, P-loop containing, Lalb_Chr09g0323971	RAB family, protein trafficking	24.5 (3.4 × 10^−06^)	25.6 (8.2 × 10^−07^)	24.8 (2.8 × 10^−06^)
Flowering time control protein FCA, Lalb_Chr13g0298581	RNA binding	24.0 (6.0 × 10^−06^)	24.4 (3.5 × 10^−06^)	21.9 (6.0 × 10^−05^)
Small GTPase superfamily, P-loop containing, Lalb_Chr23g0266071	Rac-like GTP-binding protein, signal transduction	23.9 (6.4 × 10^−06^)	15.2 (2.4 × 10^−02^)	21.7 (6.8 × 10^−05^)
Transcription factor WRKY family, Lalb_Chr18g0048721	Similar to WRKY2, regulation of gene expression	23.9 (4.7 × 10^−07^)	23.2 (8.6 × 10^−07^)	24.6 (2.0 × 10 ^−07^)
Histone acetyltransferase chromatin regulator PHD, Lalb_Chr06g0162921	PHD (plant homeodomain) zinc fingers, regulation gene expression	23.3 (1.4 × 10^−05^)	23.9 (6.8 × 10^−06^)	24.2 (5.8 × 10^−06^)
Smr domain-containing protein, Lalb_Chr14g0375461	Polyadenylate-binding protein-interacting; post-transcriptional regulation	23.2 (1.5 × 10^−05^)	19.6 (5.2 × 10^−04^)	22.7 (2.9 × 10^−05^)
RING-type E3 ubiquitin transferase, Lalb_Chr11g0068041	Ubiquitination of proteins	22.7 (2.6 × 10^−05^)	21.4 (8.8 × 10^−05^)	23.3 (1.5 × 10^−05^)
Winged helix-turn-helix DNA-binding domain, Lalb_Chr20g0109001	Regulation of gene expression	22.1 (5.0 × 10^−05^)	21.5 (7.9 × 10^−05^)	21.1 (1.2 × 10^−04^)
Zinc finger, RanBP2-type, Lalb_Chr15g0081991	Interacting with RNA or proteins	21.9 (5.9 × 10^−05^)	19.7 (4.8 × 10^−04^)	22.0 (5.2 × 10^−05^)
V-type proton ATPase subunit a, Lalb_Chr01g0004101	Vacuolar transport of ions and metabolites	21.8 (2.0 × 10^−15^)	19.8 (1.3 × 10^−12^)	20.7 (1.1 × 10^−13^)
Staygreen protein, Lalb_Chr05g0214731	Senescence-induced functions in plastids	21.7 (7.2 × 10^−05^)	20.5 (2.2 × 10^−04^)	21.2 (1.1 × 10^−04^)
Lon protease homolog 2, peroxisomal, Lalb_Chr05g0220701/	Degradation of oxidized proteins	21.5 (8.5 × 10^−05^)	23.0 (1.7 × 10^−05^)	21.1 (1.2 × 10^−04^)
ATP-dependent DNA helicase, Lalb_Chr16g0380641	Unwinding DNA or RNA in replication or transcription	21.4 (9.1 × 10^−05^)	21.7 (7.1 × 10^−05^)	20.7 (1.7 × 10^−04^)
Target of Myb protein, Lalb_Chr07g0184041	Regulated by Myb transcription factor	21.3 (9.8 × 10^−05^)	19.7 (4.8 × 10^−04^)	22.4 (3.8 × 10^−05^)
GBF-interacting protein, Lalb_Chr10g0107481	Enhance binding of G-box binding transcription factors to DNA	21.3 (9.8 × 10^−11^)	18.7 (2.7 × 10^−08^)	19.5 (7.5 × 10^−09^)
Vacuolar protein sorting-associated protein, Lalb_Chr13g0298831/	Sorting and packaging of vacuolar proteins into transport vesicles	20.9 (3.0 × 10^−11^)	19.6 (4.4 × 10^−10^	20.3 (1.3 × 10^−10^)
Transcription factor MYB-HB-like, Lalb_Chr19g0132831	Regulation of gene expression	20.9 (1.5 × 10^−04^)	21.7 (6.5 × 10^−05^	20.7 (1.7 × 10^−04^)
SPOROCYTELESS-like EAR-containing protein, Lalb_Chr25g0286021	Transcriptional repressor	20.6 (1.9 × 10^−04^)	20.3 (2.7 × 10^−04^)	20.2 (2.8 × 10^−04^)
K domain-containing protein, Lalb_Chr09g0320521	Nucleic acid-binding protein	20.6 (3.9 × 10^−11^)	19.8 (2.3 × 10^−10^)	22.0 (9.9 × 10^−13^)
Uncharacterized protein, Lalb_Chr17g0346011	Unknown	20.6 (3.3 × 10^−05^)	18.9 (1.8 × 10^−04^)	20.0 (6.2 × 10^−05^)
F-box domain-containing protein, Lalb_Chr03g0043711	Ubiquitination of proteins	20.6 (2.1 × 10^−04^)	20.6 (2.0 × 10^−04^	21.6 (7.5 × 10^−05^)
Transcription factor AP2-EREBP family, Lalb_Chr18g0059441	Regulation of gene expression	20.4 (4.1 × 10^−05^)	19.5 (9.6 × 10^−05^)	18.7 (2.5 × 10^−04^)
Bicarbonate transporter, Lalb_Chr18g0058431	Active membrane transporter	20.3 (2.6 × 10^−04^)	23.1 (1.6 × 10^−05^)	21.4 (9.3 × 10^−05^)
Major facilitator, sugar transporter, Lalb_Chr02g0144351	Transport of sugar and other substances across membrane	20.3 (2.7 × 10^−04^)	21.6 (7.5 × 10^−05^)	23.1 (1.8 × 10^−05^)
E3 SUMO protein ligase, Lalb_Chr09g0320801	Bridging SUMO and ubiquitin signaling pathways	20.3 (2.8 × 10^−04^)	20.8 (1.6 × 10^−04^)	24.0 (7.5 × 10^−06^)
Invertase, Lalb_Chr21g0305861	Cleaves sucrose into glucose and fructose	20.1 (3.2 × 10^−04^)	20.4 (2.3 × 10^−04^)	21.2 (1.2 × 10^−04^)
C2 domain-containing protein, Lalb_Chr17g0347941	Various functions, including signal transduction and membrane trafficking	20.0 (8.5 × 10^−05^)	20.8 (3.5 × 10^−05^)	19.7 (1.1 × 10^−04^)
UDP-glucuronate 4-epimerase, Lalb_Chr19g0127311	Conversion of UDP-glucuronate to UDP-D-galacturonate	19.8 (8.7 × 10^−07^)	19.7 (8.2 × 10^−07^)	19.6 (1.4 × 10^−06^)
Uncharacterized protein, Lalb_Chr01g0011871	Unknown	19.7 (8.3 × 10^−17^)	21.5 (1.7 × 10^−20^)	21.8 (2.6 × 10^−21^)
Plant organelle RNA recognition domain-containing protein, Lalb_Chr20g0111061	RNA-binding	19.5 (5.4 × 10^−4^)	5.4 × 10^−04^ (1.3 × 10^−03^)	21.8 (6.3 × 10^−5^)

**Table 5:** Promoter motif enrichment in genes up-regulated in response to sucrose.

Most Similar Motif in Arabidopsis (on Top) Compared to Enriched Motif (Bottom Logo)	Protein in Arabidopsis That May Bind to Motif (Similarity *p*-Value)	Number of Promoters Containing Motif	
		43 genes up-regulated at all timepoints *	43 genes down-regulated at all timepoints
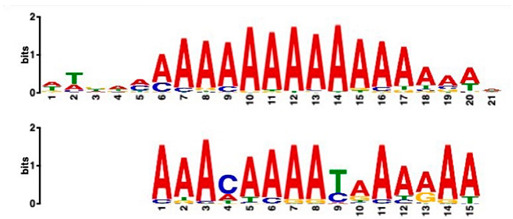	DNA-binding protein REM (B3 DNA binding domain) (2.28 × 10^−6^)	27/43 (enriched, *p*-value 2.7 × 10^−8^)	3/43
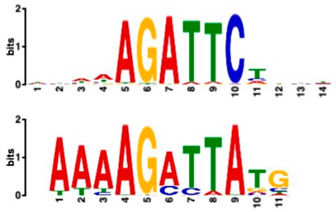	Transcription factor HHO6, probably involved in phosphate signaling in roots (6.7 × 10^−4^)	36/43 (enriched, *p*-value 6.6 × 10^−6^)	15/43
		20 genes up-regulated at all timepoints	20 genes ONLY up-regulated at t20
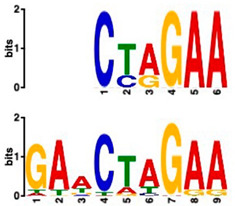	Heat shock factor (transcription factor) (4.77 × 10^−4^)	19/20 (enriched, *p*-value 2 × 10^−7^)	3/20
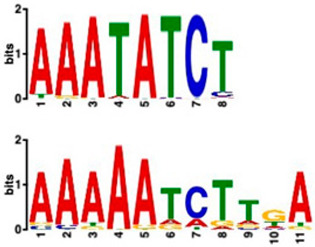	Transcription factor CCA1 (MYB-related transcription factor) (6.67 × 10^−4^)	18/20 (enriched, *p*-value 2.6 × 10^−7^)	2/20
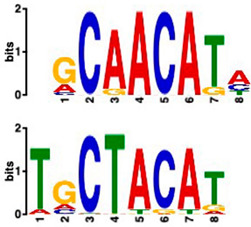	AP2/ERF and B3 domain-containing transcription factor (3.44 × 10^−3^)	1/20	15/20 (enriched, *p*-value 5 × 10^−6^)
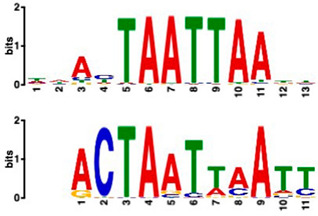	Zinc-finger homeodomain transcription factor (4.65 × 10^−7^)	0/20	13/20 (enriched, *p*-value 6.4 × 10^−6^)
		10 genes induced in cluster root development (Figure 3)	10 genes not induced in cluster root development
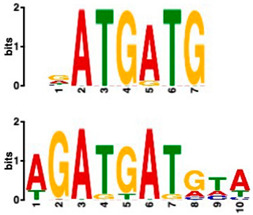	MADS-box transcription factor (7.84 × 10^−6^)	10/10 (enriched, *p*-value 5.4 × 10^−6^)	0/10

* column subheaders are shown in bold.

**Table 6:** Count of up-regulated kinases and phosphatases, compared to the untreated control (0 min).

Duration of Sucrose Exposure	Count of Up-Regulated *		Ratio of Kinase/Phosphatase
	Kinases	Phosphatases	
20 min	17	4	4.25
40 min	4	3	1.33
80 min	3	3	1

* (log2FC ≥ 1.5, padj ≤ 0.05).

## Data Availability

The raw data and processed FPKM data for all samples of this RNA-seq experiment are openly accessible through the NCBI GEO Series accession number GSE283515 (https://www.ncbi.nlm.nih.gov/geo/query/acc.cgi?acc= GSE283515, submitted on 4 December 2024).

## Supplementary Materials

The following supporting information can be downloaded at: https://www.mdpi.com/article/10.3390/plants14030381/s1, Table S1: Raw_data_for_Figure 2, Table S2: Raw_data_for_Figure 3, Table S3: Raw_data_for_Figure 4.
